# Supplementary material for: Early Intervention of Pulmonary Rehabilitation for Fibrotic Interstitial Lung Disease Is a Favorable Factor for Short-Term Improvement in Health-Related Quality of Life
Source: J Clin Med. 2021 Jul 16;10(14):3153. doi: 10.3390/jcm10143153 (PMC8305639; doi:10.3390/jcm10143153)
Supplement: Supplementary file 1 [file jcm-10-03153-s001.zip › jcm-1285813-supplementary.pdf]

Supplementary Materials

Table S1. Differences between baseline values and 6MWD and SGRQ total score.

		6MWD, m	*P* value	SGRQ total	*P* value
Gender	Male	307.0 (243.8–281.4)	0.018*	46.9 (33.7–68.7)	0.17
	Female	232.5 (193.8–249.8)		57.8 (54.1–67.3)	
FILD subtype	IPF	280.0 (245.0–318.0)	0.012*	53.0 (38.1–65.3)	0.53
	Non-IPF	225.0 (192.5–241.5)		61.4 (43.0–68.4)	
mMRC scale	0–2	311.5 (270.0–335.0)	0.022*	38.0 (31.9–50.0)	0.0050*
	3–4	244.0 (217.2–268.5)		65.0 (53.0–77.2)	
Therapy at baseline					
Corticosteroid	With	264.0 (243.0–315.0)	0.59	70.9 (43.6–77.3)	0.21
	Without	268.0 (215.0–313.0)		53.0 (37.7–55.9)	
Anti-fibrotic agent	With	306.0 (254.0–330.5)	0.35	53.0 (38.2–73.2)	0.86
	Without	255.0 (225.0–308.0)		53.0 (38.0–65.0)	
LTOT	With	245.0 (195.0–318.0)	0.57	70.9 (53.2–77.3)	0.020*
	Without	270.0 (240.0–311.5)		46.0 (35.0–55.9)	

Data are presented as median (interquartile range). *P < 0.05.

FILD, fibrosing interstitial lung disease; mMRC, modified Medical Research Council; 6MWD, 6-minute walk distance;

LTOT, long-term oxygen therapy; SGRQ, St. George's Respiratory Questionnaire.

Table S2. Differences between baseline values and response to pulmonary rehabilitation

		Δ6MWD, m	*P* value	ΔSGRQ total	*P* value
Gender	Male	8.5 (−11.8–50.0)	0.63	−2.6 (−6.1–2.4)	0.29
	Female	8.5 (−6.3–22.3)		3.8 (−1.8–7.2)	
FILD subtype	IPF	16.0 (−10.0–50.0)	0.47	−1.6 (−5.0–3.5)	0.71
	Non–IPF	6.0 (−2.5–11.5)		−3.6 (−9.6–4.0)	
mMRC scale	0–2	13.5 (3.8–56.0)	0.12	−2.8 (−4.4–2.4)	0.85
	3–4	2.5 (−30.3–32.5)		1.3 (−7.7–5.9)	
Therapy at baseline					
Corticosteroid	With	5.0 (−43.0–50.0)	0.69	−3.0 (−5.0–0.4)	0.55

	Without	12.0 (−2.5-37.0)		0.1 (−5.5–3.9)	
Anti–fibrotic agent	With	40.0 (10.5-62.0)	0.087	−1.2 (−4.4–2.4)	0.84
	Without	5.0 (−10.0-32.0)		−2.3 (−8.4–7.2)	
LTOT	With	16.0 (−7.0-50.0)	0.57	−3.6 (−6.1–−1.1)	0.50
	Without	5.0 (−7.5-37.0)		−0.3 (−4.3–3.9)	

Data are presented as median (interquartile range).

FILD, fibrosing interstitial lung disease; mMRC, modified Medical Research Council; LTOT, long-term oxygen therapy; 6MWD, 6-minute walk distance; SGRQ, St. George's Respiratory Questionnaire.
